# Quantifying the effect of swab pool size on the detection of influenza A viruses in broiler chickens and its implications for surveillance

**DOI:** 10.1186/s12917-018-1602-1

**Published:** 2018-09-03

**Authors:** Amos Ssematimba, Sasidhar Malladi, Peter J. Bonney, Cristian Flores-Figueroa, Jeannette Muñoz-Aguayo, David A. Halvorson, Carol J. Cardona

**Affiliations:** 1 Secure Food Systems Team, University of Minnesota, Veterinary and Biomedical Sciences, 301C Veterinary Science Building, 1971 Commonwealth Avenue, Saint Paul, MN 55108 USA; 2 Mid Central Research and Outreach Center, 1802 18th St NE, Willmar, MN 56201 USA; 3 Department of Mathematics, Faculty of Science, Gulu University, P.O. Box 166, Gulu, Uganda

**Keywords:** Influenza A virus, Surveillance, Real-time polymerase chain reaction, Antigen detection, Sample pooling, Lateral flow immunoassays

## Abstract

**Background:**

Timely diagnosis of influenza A virus infections is critical for outbreak control. Due to their rapidity and other logistical advantages, lateral flow immunoassays can support influenza A virus surveillance programs and here, their field performance was proactively assessed.

The performance of real-time polymerase chain reaction and two lateral flow immunoassay kits (FluDETECT and VetScan) in detecting low pathogenicity influenza A virus in oropharyngeal swab samples from experimentally inoculated broiler chickens was evaluated and at a flock-level, different testing scenarios were analyzed.

**Results:**

For real-time polymerase chain reaction positive individual-swabs, FluDETECT respectively detected 37% and 58% for the H5 and H7 LPAIV compared to 28% and 42% for VetScan. The mean virus titer in H7 samples was higher than for H5 samples. For real-time polymerase chain reaction positive pooled swabs (containing one positive), detections by FluDETECT were significantly higher in the combined 5- and 6-swab samples compared to 11-swab samples. FluDETECT detected 58%, 55.1% and 44.9% for the H7 subtype and 28.3%, 34.0% and 24.6% for the H5 in pools of 5, 6 and 11 respectively.

In our testing scenario analysis, at low flock-level LPAIV infection prevalence, testing pools of 11 detected slightly more infections while at higher prevalence, testing pools of 5 or 6 performed better. For highly pathogenic avian influenza virus, testing pools of 11 (versus 5 or 6) detected up to 5% more infections under the assumption of similar sensitivity across pools and detected less by 3% when its sensitivity was assumed to be lower.

**Conclusions:**

Much as pooling a bigger number of swab samples increases the chances of having a positive swab included in the sample to be tested, this study’s outcomes indicate that this practice may actually reduce the chances of detecting the virus since it may result into lowering the virus titer of the pooled sample. Further analysis on whether having more than one positive swab in a pooled sample would result in increased sensitivity for low pathogenicity avian influenza virus is needed.

## Background

Influenza type A viral (IAV) infections in poultry may either be of low or high pathogenicity. IAV evolve rapidly [1] and occasionally, once introduced into poultry, low pathogenicity avian influenza (LPAI) viruses (LPAIV) of the H5 or H7 subtypes from the wild bird reservoir can directly evolve into highly pathogenic avian influenza (HPAI) viruses (HPAIV) [2, 3]. Although infections by both viruses largely have the same host range, they cause different diseases in terms of virulence [4, 5] and hence result in different needs for control and surveillance. For example, while infections with HPAIV are associated with high morbidity and mortality, LPAI is usually asymptomatic or responsible for a mild respiratory disease that may cause a reduction in egg production or moderately increased mortality [4]. Unlike LPAI where the infection can be detected via the presence of either virus or antibodies since infected birds mostly recover, almost no HPAIV infected poultry will survive [5] and infection is mostly detected via testing sick or dead birds for virus. Also, during outbreaks, HPAIV has been shown to easily spread by proximity [3] while LPAIV often spreads through contact networks [6].

The essential components for avian influenza control are education of flock owners and workers, biosecurity, prevention, surveillance [6], and vaccination (depending on the country). There are both passive and active surveillance systems in place in most countries to ensure early detection of IAV circulation in poultry with the former well suited for HPAIV detection due to its clear clinical manifestation. Laboratory testing using sensitive testing procedures is key for an efficient surveillance system. Although polymerase chain reaction (PCR) is extensively used during IAV outbreaks [7], the shorter turnaround time and other logistical advantages render lateral flow immunoassays (LFI) desirable for field screening; even though LFI have a lower sensitivity relative to PCR or virus isolation [8, 9].

Individual test characteristics, sample collection and composition all play a vital role in determining the efficacy of IAV active surveillance systems. Precisely, given a pooled sample with at least one positive swab, the probability that the sample tests positive depends on test characteristics, the virus titer in the positive swab, the volume of the diluent and on the number of negative swabs it is pooled with. Swab-pooling has been shown to allow a single test for multiple samples. In other words, not only does swab-pooling increase testing throughput, it also reduces the number of tests needed to meet sample size requirements and consequently the testing costs. However, due to dilution, pooling may also significantly reduce test sensitivity and lead to missing low prevalence infections [10, 11].

Recommendations to improve the sensitivity of LFI in IAV surveillance have included testing large numbers of samples before confirming the absence of the virus and avoiding excessive dilution of samples by pooling [9]. Indeed, Loth et al. [12] demonstrated that as the percentage of birds in the flock shedding virus increased, the number of animals to be tested to attain a given flock sensitivity was reduced. Thus, proactive evaluation of LFI performance under different sampling and surveillance protocols is important.

Since LFI target the type A influenza virus nucleoprotein, they are suitable for any type A influenza virus [9, 13, 14]. Here, using H5N2 and H7N2 low pathogenicity influenza A viruses as models, we evaluated the impact pooling one positive swab with 4 or 5 versus 10 negative swabs on the ability of LFI kits to detect IAV in pools and also to compare individual tests’ analytical sensitivities on single swabs.

We compared the effectiveness of two LFI kits, namely, FluDETECT (Synbiotics Corporation, San Diego, California, USA) and VetScan (Abaxis, Inc., Union City, California, USA), in detecting two influenza A viruses and assess the effect of sample pooling on test performance. Lastly, we also compared the effectiveness of proposed antigen capture-based testing protocols involving different swab-pooling schemes during IAV field surveillance.

## Methods

The studies described were fully compliant with the University of Minnesota Institutional Animal Care and Use and the Institutional Biosafety Committee policies and were conducted under protocols 1409-31802A and 1610-34222H respectively.

### Animals

Three hundred thirty (330) eleven-day old commercial broiler chickens (free of maternally derived antibodies against IAV) purchased from a commercial farm were transported to the Animal Resources Unit at the University of Minnesota. One hundred twenty-six (126) of the chicks were housed in six (6) isolator units, i.e., 21 birds/isolator under Biosafety Level 2 conditions while 204 were kept in Biosafety Level 1 floor housing. All birds were provided standard broiler grow ration and clean water which were provided ad libitum.

### Challenge study

Six isolators were randomly assigned into two challenge groups. Birds in three isolators (*n* = 63 birds) were inoculated intranasally with 10^6^ EID_50_/ml of H5 LPAIV (Chicken/PA/13609/93, H5N2), while the birds in the other three isolators (n = 63 birds) were inoculated intranasally with 10^6^ EID_50_/ml of H7 LPAIV (Guinea hen/MA/148081/2002, H7N2). Re-titration from one dose of the inocula yielded titers of 10^5.4^ EID_50_/ml and 10^5.8^ EID_50_/ml for the H5 and H7 viruses respectively. Bird comfort and clinical signs were observed and recorded daily. Dead birds (2 total) were recorded and removed but were used for the swab samples for that day. At the end of the experiment, all remaining animals were euthanized by inhalation of carbon dioxide.

Oropharyngeal (OP) swab samples were taken from all birds on days 5–8 post-challenge using sterile polyester tipped applicators (Puritan Medical Products Company LLC, Guilford, Maine, USA). One hundred thirty-three (69 from H5-inoculated group and 64 from the H7-inoculated group) individual swab samples were used for direct comparison of RT-PCR, FluDETECT and VetScan tests. For the pooled sample analysis, 298 samples from the H5-inoculated group and 227 samples from the H7 inoculated group were used. One swab was placed into a 4-, 5- or 10- pool of swabs from uninoculated birds in brain heart infusion (BHI) or individually into a tube with 1.0 ml of BHI broth. All tubes were kept on ice until they were transported to the laboratory where they were stored at − 80 °C until they were tested.

### Negative swab collection to create pool

Twice daily, uninoculated birds held in a floor unit were swabbed. The swabs were placed into pools of 4, 5, or 10. The pools of 4 and 5 swabs were placed into 3 ml of BHI and the 10-swab pool was placed into 5.5 ml of BHI. The pools were kept on ice until they were refrigerated in the laboratory.

### Antigen detection testing

Two commercially available lateral flow immunoassay kits FluDETECT and VetScan were used for the detection of Influenza A virus in single swabs and only FluDETECT was used for the detection of IAV in pooled swab samples. For both tests, the manufacturer’s instructions were followed.

### RT-PCR

RNA was extracted from 50 μl of each sample with the MagMAX-96 Viral RNA Isolation Kit (Ambion) using MagMAX Express magnetic particle processor (Applied Biosystems) according to manufacturer’s instructions. For IAV RT-PCR, each 25 μl reaction contained: 8 μl RNA, 0.83 μl of ultra-pure water, 12.5 μl of 2X buffer, 0.25 μl of 20 μM of each primer, 0.25 μl of 6 μM probe, 1.67 μl detection enhancer, and 1.0 μl 25X enzyme mix AgPath-ID ™ One-Step RT-PCR Kit (Ambion).Cycling conditions on Stratagene Mx 3005P were: 45 °C for 10 min, 95 °C for 10 min, 45 cycles of 94 °C for 15 s, 60 °C for 45 s [15, 16].

### Standard curve generation

Each of the challenge viruses was titered in 9–11 day old embryonating chicken eggs (5 eggs were inoculated per dilution) following standard procedures [17]. Viral titers were calculated for each isolate using the Reed and Muench formula. The H5 titer was 1.49 * 10^8^/ml while the H7 titer was 4.93 * 10^9^/ml. Viral stocks were log fold diluted and tested with RT-PCR. CT values were plotted against virus titers to create a standard curve. The standard curve was used to calculate viral particle numbers based on quantitative RT-PCR CT values.

### Data analysis

Data were transferred to a spreadsheet (Excel, Microsoft Corporation, Redmond, WA, USA) and the Statistical software R [18] was used to perform the analyses. For the single-swab samples, the proportions positive in both lateral flow immunoassays as well as the mean and lowest virus titer detected were determined. For the pooled-swab samples, the three swab pooling schemes were evaluated both within and between subtypes to assess the effect of swab-pooling on the performance of FluDETECT. Its ability to detect the virus in pools of 11 was compared with that in individual pools of 5 or 6 and in combined pools of 5 or 6 for separate H5 and H7 subtypes as well as for merged H5 and H7 sample groups. Only samples with CT ≤ 35 were considered RT-PCR positive [11, 19] and in the comparison of virus positive proportions, Fisher’s exact test at significance level of *p* ≤ 0.05 was used [20].

### Influenza A virus testing scenario analyses

Using FluDETECT as a model test, we evaluated the potential use of LFI-based testing protocols as a surveillance tool for IAV infections in poultry. As application examples, we assessed the detection abilities of LFI alone for LPAIV and in combination with a 0.3% daily mortality trigger and targeted-sampling of only dead birds for HPAIV.

We performed 6000 simulations using a re-parameterized version of the mathematical model described by [21]. For IAV transmission dynamics in a broiler flock, the variables and parameters used are presented in Table 1. The outputs of interest at different days post infection (dpi) were mean infection prevalence in the flock and the mean percentage of outbreaks that are detected. The assessed testing protocols involved testing three OP swab samples comprising of either 5, 6 or 11 swabs.

**Table 1:** Summary of the variables and parameter values or distributions used in the preliminary IAV testing scenario analyses in a broiler chicken flock

Parameter name	Parameter description	Distribution	
		LPAIV	HPAIV
Infectious period distribution	Length of the infectious period for IAV in chickens	Gamma: shape = 8.1388, scale = 0.9596	Weibull: shape = 1.965, scale = 2.90
Latent period distribution	Length of the latent period for IAV in chickens	Gamma: shape = 0.8248, scale = 0.4446	Gamma: shape = 0.89, scale = 0.7145
Adequate contact rate	The number of contacts per unit time that a broiler has with other broilers that are adequate to transmit IAV	Uniform (0.69–0.77)	PERT: minimum = 2.5, mode = 4.77, maximum = 9.0
Disease mortality	Proportion of IAV infected birds that succumbs to disease	Fixed: 0.5%	Fixed: 100%
Flock size	The number of broiler chickens per house	Log-normal: log mean = 10.0212, log SD = 0.3883 truncated at 13,000 and 50,000 birds	Same as for LPAIV
FluDETECT positive proportions	The fraction of positives detected by FluDETECT in different pooling schemes	Fixed: 58.0%, 55.1% and 44.9% for pools of 5, 6 and 11 respectively	Scenario 1: 71% [12], Scenario 2: 71%, 68.1%, 57.9% for pools 5, 6 and 11 respectively

For the model, disease-induced mortality [22, 23], distributions for the adequate contact rate [5, 24–28], and latent [5, 26, 29–31] and infectious [32–36] period durations were estimated from the data in the indicated literature. FluDETECT positive proportions for LPAIV were determined from this study. For HPAI, since no testing data was available in our desired format, we used the mean estimate from a study on HPAI H5N1 virus in chickens [12] with the assumption that FluDETECT sensitivity does not vary across sample pool sizes (Scenario 1) or it varies by similar magnitudes as for LPAIV pools (Scenario 2).

## Results

All 330 chickens were free of maternally derived antibodies against IAV at the start of the experiment and two birds died during the course. They were removed upon being recorded and were used for the swab samples for that day.

### Descriptive summary of test results of the single-swab samples

A total of 133 (i.e., 69 for the H5 and 64 for the H7 virus) individual swabs were tested using all three tests. By our definition of an RT-PCR positive sample (i.e., with CT ≤ 35), we obtained 43 (62.3%) RT-PCR positive samples with a mean titer of 10^2.43^EID_50_/ml for the H5 subtype and 62 (96.9%) with mean titer of 10^3.73^EID_50_/ml for the H7 subtype. The highest virus titers detected were 10^3.18^ and 10^4.7^ EID_50_/ml for the H5 and H7 viruses respectively. Table 2 presents the summary of single-swab samples testing results for both LFI for only the RT-PCR positive samples i.e., those with CT ≤ 35.

**Table 2:** Summary results on positive proportions and virus titer present for the single-swab samples for only samples with CT ≤ 35 totaling *n* = 43 and 62 for H5 and H7 viruses respectively^a^

Test	Subtype	Number positive (%)	Mean virus titer for LFI detected samples in EID_50_/ml	Lowest virus titer for LFI detected samples in EID_50_/ml
FluDETECT	H5	16 (37%)	10^2.98^	10^2.32^
	H7	36 (58%)	10^3.62^	10^2.65^
VetScan	H5	12 (28%)	10^3.03^	10^2.39^
	H7	26 (42%)	10^3.73^	10^2.65^

^a^For all PCR positive single-swab samples (i.e., those with CT ≤ 35), the mean virus titer was 10^2.43^EID_50_/ml and 10^3.73^EID_50_/ml for the H5 and H7 viruses respectively

### Descriptive summary of test results of the pooled-swab samples

**Fig. 1:**
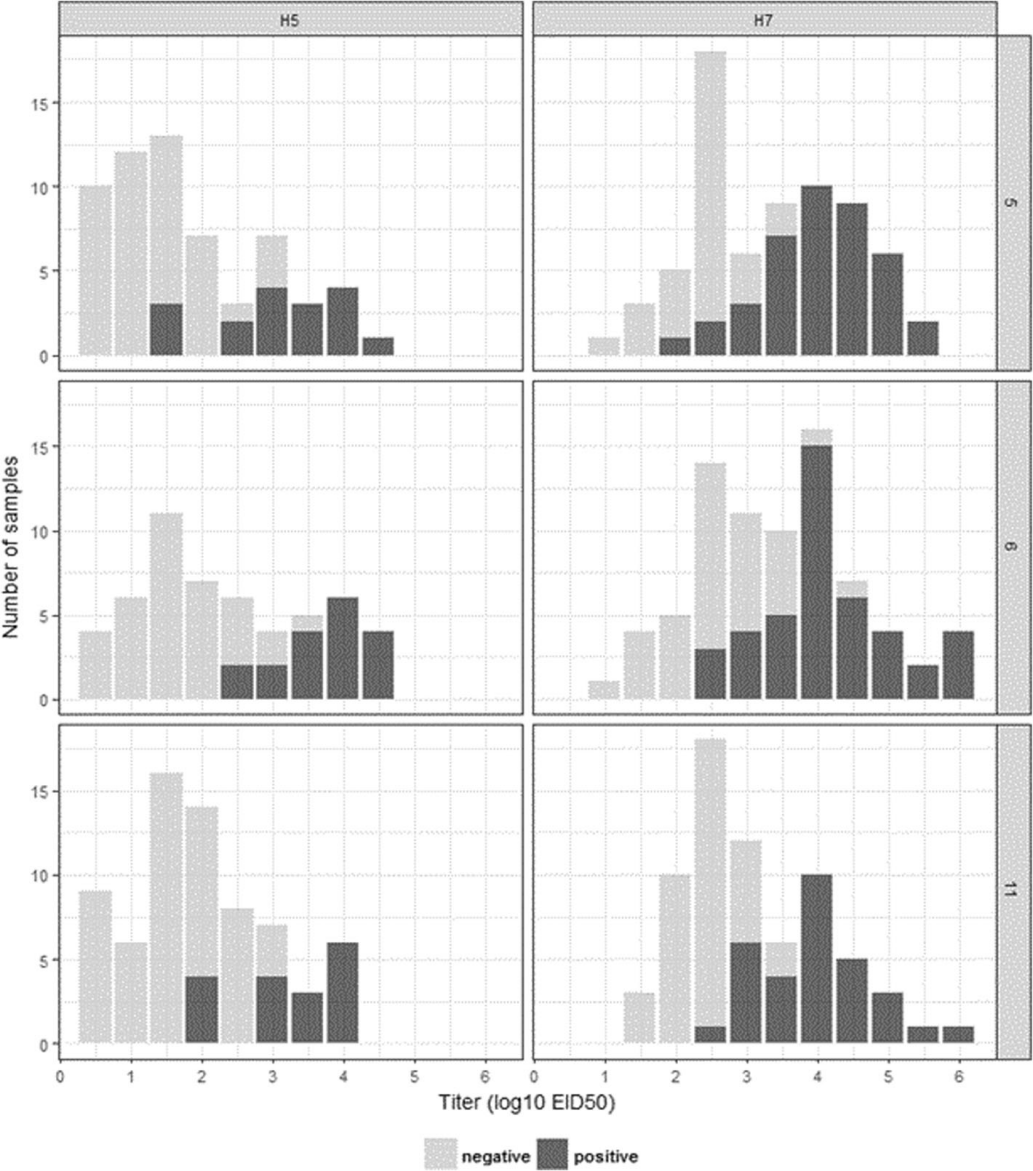
Distribution of samples by virus titer (EID_50_ log_10_/ml) present and FluDETECT test results. Left panels depict results for the H5 samples and the right panels are for H7 samples. The results for pools of 5, 6 and 11 are presented from top to bottom respectively. Note that these are results for samples that had CT ≤ 35 whose individual totals are indicated in Table 3

**Fig. 2:**
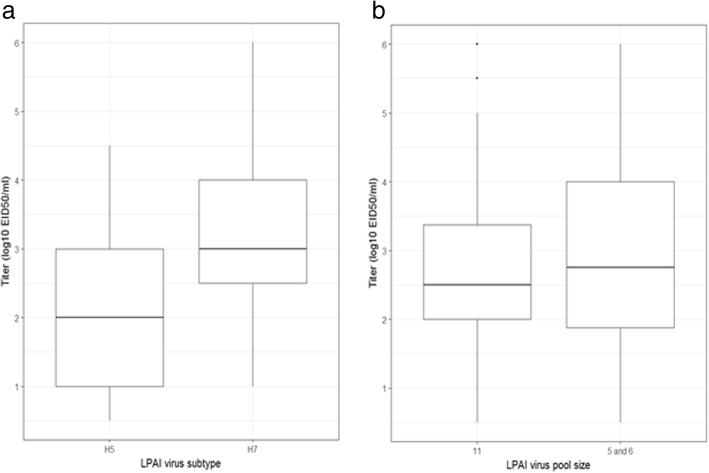
Virus titer (EID_50_ log_10_/ml) distribution of the pooled-swab samples. Panel **a** depicts a comparison of virus titers by the subtype for all pool sizes combined and **b** depicts a comparison of virus titers by pool sizes for combined subtypes. The boxplots depict the minimum, 25th percentile, median, 75th percentile and the maximum virus titer present. These are results for samples that had CT value ≤35

The collected swabs were differently pooled into 525 samples (of which 298 and 227 were respectively of H5 and H7 subtype) for testing and further analysis. Figures 1 and 2 as well as Table 3 present the results on the proportions of samples that were positive in antigen detection and RT-PCR as well as their calculated virus titer.

**Table 3:** Summary results on positive proportions and virus titer present in the pooled-swab samples; unless stated otherwise, the results are for samples that are considered RT-PCR positive i.e., those with CT ≤ 35

Subtype	Pool size	Number RT-PCR positive/Total (% ^a^)	Mean titer for PCR positive samples in EID_50_/ml	Highest titer in EID_50_/ml	Number FluDETECT positive (% ^b^)	Mean titer for FluDETECT positive in EID_50_/ml
H5	5	60/98 (61.2%)	10^1.60^	10^4.26^	17 (28.3%)	10^2.86^
	6	53/91 (58.2%)	10^2.09^	10^4.32^	18 (34.0%)	10^3.46^
	11	69/109 (63.3%)	10^1.78^	10^4.00^	17 (24.6%)	10^2.99^
H7	5	69/74 (93.2%)	10^3.13^	10^5.33^	40 (58.0%)	10^3.87^
	6	78/80 (97.5%)	10^3.19^	10^5.94^	43 (55.1%)	10^3.92^
	11	69/73 (94.5%)	10^2.87^	10^5.55^	31 (44.9%)	10^3.73^

^a^As a percentage of all samples of the specific virus subtype and pool size

^b^As a percentage of samples with CT ≤ 35

In Table 3, we observe that, of all tested samples of a given subtype and pool size, RT-PCR positive proportions were up to 97.5% for the H7 pooled samples (observed in pools of 6) while the highest in H5 samples was 63.3% observed in pools of 11. We observe from Fig. 1 that there were fewer FluDETECT positive pooled samples of the H5 virus that the H7 across all pool sizes. Figure 2 summarizes the distribution of virus titer for all RT-PCR positive samples and indicates that, although not tested for statistical significance, the H7 samples had a relatively higher virus titer overall. We observe (Table 3) that, for RT-PCR positive samples, when compared within subtype, the highest proportion of H5 FluDETECT positive samples was 34%, which was estimated for pools of 6 and it was 9.4% more than the least estimate (obtained for the pools of 11). Similarly, for the H7 subtype, the highest proportion was 58% estimated for the pools of 5, and it was 13.1% more than the least estimate (corresponding to pools of 11). Between subtypes, for all corresponding pool sizes, the H7 subtype had higher FluDETECT positive proportions than H5, and the biggest difference was 29.7%, observed in the pools of 5. The mean titer of RT-PCR positive samples was also more than one log_10_ higher for H7 subtype samples for all corresponding pool sizes (Table 3).

### Performance of FluDETECT under different sample pooling schemes

Results from the statistical comparisons of the positive proportions to assess the performance of FluDETECT in pools of 5 and/or 6 versus 11 for PCR positive samples (CT ≤ 35) are presented in Table 4. We observe that pooling 11 swabs resulted in a significantly lower proportion of samples that test positive using LFI compared to pools of 5 or 6 for all RT-PCR positive H5 and H7 samples combined (*p* = 0.026) and pools of 6 for all RT-PCR positive H7 samples (*p* = 0.033). The other noteworthy comparisons (i.e., those significant at *p* ≤ 0.1) in which pools of 11 had lower positive proportions were between pools of 11 and pools of 5 for all PCR positive H5 and H7 samples combined (*p* = 0.074) and pools of 5 or 6 (*p* = 0.075) and pools of 5 (*p* = 0.086) for only H7 RT-PCR positive samples.

**Table 4:** Pairwise comparison of FluDETECT positive sample proportions for different sample pooling schemes for RT-PCR positive samples (i.e., those with CT ≤ 35) using one-sided Fisher’s exact test

Subtype	Pool size	Fraction FluDETECT positive (%): Fraction for 11-swab pools (%)^a^	Fisher’s test: AC positive greater for 11-swab pools
H5 and H7	5 or 6	118/260 (45.4%): 48/138 (34.8%)	0.026
	5	57/129 (44.2%): 48/138 (34.8%)	0.074
	6	61/131 (46.6%): 48/138 (34.8%)	0.033
H5	5 or 6	35/113 (31.0%): 17/69 (24.6%)	0.228
	5	17/60 (28.3%): 17/69 (24.6%)	0.391
	6	18/53 (34.0%): 17/69 (24.6%)	0.177
H7	5 or 6	83/147 (56.5%): 31/69 (44.9%)	0.075
	5	40/69 (58.0%): 31/69 (44.9%)	0.086
	6	43/78 (55.1%): 31/69 (44.9%)	0.142

^a^In parentheses is the FluDETECT positive fraction for pools of size 11 for that subtype grouping

### IAV testing scenario analysis outcomes

**Fig. 3:**
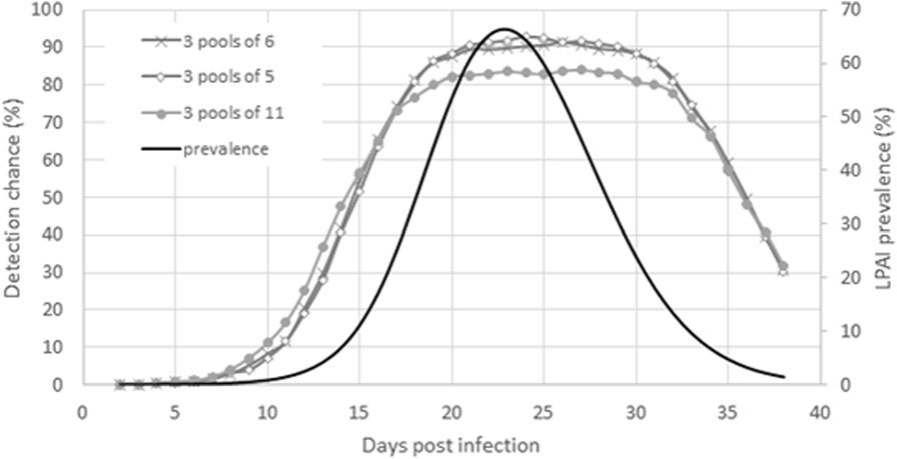
LPAIV testing scenario analysis results with lateral flow immunoassay FluDETECT. The primary and secondary y-axes present outcomes on for the different pooled sample compositions and LPAI infection prevalence at different days post infection of a broiler flock. The assessed scenarios include testing 3 pools each of 5, 6 and 11 pooled samples on different days post infection. The test sensitivities used are estimated in this study for when only one swab in the pooled swab sample is positive

For LPAIV (Fig. 3), there was a negligible difference in test outcomes for pools of 5 and 6 swabs throughout the simulated period (38 days). Compared to pools of 5 and 6, testing pools of 11 resulted in a slightly higher percentage of detections between 5 and 15 dpi and after 36 dpi when infection prevalence was low and the trend is reversed on the other days when prevalence was high.

For HPAIV (Table 5), we found that all evaluated pooling protocols achieved 100% detection rate by 7 dpi. Since we could not test the differences for statistical significance, we use the phrase “substantive difference” to refer to situations where the difference between detected proportions is 2% or more. Within either of the scenarios assessed, the predicted detection percentages for pools of 5 and 6 swabs were in close agreement (either equal or within a 1% difference) in all but one (under scenario 1 at 2 dpi where testing pools of 6 detected 2% more cases). Otherwise, under scenario 1, substantive differences in proportion detected were observed at 2 and 3 dpi with testing pools of 11 detecting 4% and 5% more than pools of 5 and 2% and 4% more than for pools of 6 respectively. Under scenario 2, the only substantive difference was on 4 dpi where testing pools of 11 resulted in detecting 3% fewer cases than pools of 5 and 6 swabs.

**Table 5:** Predicted HPAIV detection percentage at different days post infection of a broiler flock by testing three (3) dead-bird pooled swab samples of sizes 5, 6 or 11 using lateral flow immunoassay FluDETECT with varying test sensitivities (se) together with 0.3% daily mortality trigger*

Days post flock infection	1	2	3	4	5	6	7
Flock infection prevalence (%) ^#^	0.0	0.1	0.4	2.7	13.0	32.5	48.8
1^a^							
3 pools of 5: se = 71%	6	27	64	88	96	99	100
3 pools of 6: se = 71%	5	29	65	88	96	99	100
3 pools of 11: se = 71%	6	31	69	89	97	99	100
2^b^							
3 pools of 6: se = 68.1%	5	27	65	88	97	99	100
3 pools of 11: se = 57.9%	5	26	63	85	95	99	100

^*^All results i.e. infection prevalence and detected proportions are presented as percentages

^#^The mean simulated flock size was estimated as 24,111 birds

^a^Scenario 1: assuming that LFI test sensitivity for HPAIV is the same across pool sizes

^b^Scenario 2: assuming that LFI test sensitivity for HPAIV is different across pool sizes

## Discussion

Using data from experimental infections of chickens with either H5 or H7 viruses, the performance of RT-PCR, FluDETECT and VetScan in detecting IAV was evaluated. For individual OP RT-PCR positive swabs (Table 2), FluDETECT detected 37% of H5 samples and 58% of H7 samples (9% and 16% more H5 and H7 virus PCR positive samples than VetScan). These detected proportions from clinically normal LPAIV-infected birds are lower than those reported for samples taken from HPAIV-infected sick or dead birds. Note however that even lower sensitivities (ranging from 0 to 5%) were reported for other LFI kits on individual swabs of H6N2 LPAIV [8].

In this study (conducted with LPAIV), the mean virus titers in the individual swabs detected by both LFI kits were comparable, and H7 samples had higher titers and more LFI positive proportions among RT-PCR positive samples than the H5 virus. The observed subtype effect may be due to differences in replication efficiency in the host as a result of differences in the viruses or inoculation dose used to challenge the birds [37, 38].

Our FluDETECT test results for the pooled swab samples (Table 3) revealed a subtype effect as H7 samples were found to have relatively higher virus titer and higher proportion of LFI positive samples (and consequently higher LFI sensitivity) than their H5 counterparts. On the potential effect of swab pooling, our findings for combined virus subtypes indicate that pooling up to 10 negative swabs with one positive swab reduced the viral antigen concentration and consequently the proportion of FluDETECT positive samples. This result is largely consistent with those of Ladman et al. [11]. However, a study by Spackman et al. [39] did not find differences in virus detection between a single swab from an inoculated bird, 5 swabs (1 from an inoculated bird, 4 from unexposed birds), or 11 swabs (1 from an inoculated bird and 10 from unexposed birds) by real time RT-PCR. This is not surprising because virus isolation and real time RT-PCR both have a high analytic sensitivity (i.e., low limit of detection) and hence the potential reduction in virus concentration due to pooling would have a relatively lesser impact.

On test sensitivities for HPAIV, the ability of LFI kits to detect H5N1 virus subtype has been previously assessed. For FluDETECT in particular, examples of the reported detection levels include: 55.1% infections in cloacal and combined tracheal and cloacal swabs and 76.9% in tissues from infected chickens [13]; 100% and 33.3% for doses of 10^6^ and 10^5^ EID_50_/ml in a serial dilution study [14] and, 71% (95% CI: 58–82%) in chicken samples from an outbreak [12]. These results, compared to those from this study, generally indicate that LFI kits perform better in detecting HPAIV infections. Due to data limitations, in one of our assessed scenarios, we assumed that LFI test sensitivity would vary with pool size by similar magnitudes for HPAIVs as those obtained for LPAIVs in this study. This may not necessarily be true and we recommend that studies to quantify this effect for HPAIV be performed.

For our study, since swab pooling is often practiced in the field partly as a means to cut surveillance costs [10], we compared IAV field surveillance outcomes for protocols involving testing differently pooled swab samples. We found an infection prevalence dependent swab-pooling effect on FluDETECT test performance. Specifically, for LPAIV, testing pools of 5 and 6 performed better than those of 11 at a high infection prevalence and pools of 11 performed better at low infection prevalence. For HPAIV, the evaluated protocols generally detected similar proportions or were within a 1% of each other with a few notable differences on specific days (between 2 and 4) post infection. The main observation was that, at a high and similar test sensitivity of 71%, testing pools of 11 dominates (with up to 5% more detections) and it lags by up to 4% when its sensitivity was lower compared to the pools of 5 and 6. Throughout, outcomes from testing pools of 5 and 6 were almost similar.

We hypothesize that, during disease surveillance, the implication of a negative LFI result differs between lowly and highly pathogenic avian influenza viruses due to their differences in within flock transmission dynamics and pathogenicity-related factors. Note that, unlike LPAIV, HPAIV, which is associated with mortality, targeted-sampling of only dead birds as well as the use of a mortality trigger were applied and both increased the chances of detecting IAV in a sample.

It is likely that, for LPAIV at low prevalence, pools of 11 have a greater chance of containing a positive swab and this advantage (over pools of 5 and 6) diminishes at high prevalence and the influence of sample dilution takes over [10]. Note however that there may be additional benefits of pooling more samples that we have not analyzed in this study. For example, there is generally a higher chance of including more than one positive swab in pools of 11 compared to pools of 5 and 6 and this may lead to a higher flock level test sensitivity for the 11 pool samples. In the context of LPAIV, further analysis with more swab pooling schemes, e.g., those involving more than one positive swab in the pooled samples, is needed.

## Conclusion

Despite their relatively low sensitivity, the logistical advantages of LFI support their use as part of influenza A virus outbreak management tools preferably during preliminary outbreak investigation or routine surveillance in an AI free country. Moreover, flock surveillance protocols would likely be based on multiple pooled samples. Hence, although the diagnostic sensitivity for a single pooled sample is not great, the flock level likelihood of detection can be adequate when several pooled samples are tested.

Since they are best suited for high virus titer samples, targeted sampling based on the clinical status of infected birds may improve their sensitivity. Consequently, based on factors such as high virus titer shed, high morbidity and fatality rate and fast within flock transmission, HPAIV infections may provide a better platform for LFI field deployment to determine the disease status of a flock. Note that the current diagnosis of HPAIV in control areas may only involve LFI as a supplement to PCR testing, although this may be specific to some countries depending on the regulations. Thus, our assessment generates insight into how these tests would perform when used (particularly) outside of a control area during high risk periods of HPAIV spread. We recommend that experimental and field studies evaluating additional pooling schemes involving more than one positive swabs per pooled sample be performed.

## Abbreviations

IAV: Influenza type A Virus
LPAI: Low Pathogenicity Avian Influenza
LPAIV: Low Pathogenicity Avian Influenza Virus
HPAI: Highly Pathogenic Avian Influenza
HPAIV: Highly Pathogenic Avian Influenza Virus
LFI: Lateral Flow Immunoassay
BHI: Brain Heart Infusion
PCR: Polymerase Chain Reaction
RT-PCR: Reverse Transcription Polymerase Chain Reaction
